# The development of a coding system to code planning talk within motivational interviewing

**DOI:** 10.1016/j.pec.2016.09.003

**Published:** 2017-02

**Authors:** Lauren Copeland, Rachel McNamara, Mark Kelson, Sharon Simpson

**Affiliations:** a Division of Population Medicine, Cardiff University, School of Medicine, Cardiff, UK; b South East Wales Trials Unit, Centre for Trials Research, College of Biomedical and Life Sciences, Cardiff University, Cardiff, UK; c MRC/CSO Social and Public Health Sciences Unit, University of Glasgow, 200 Renfield Street, Glasgow, UK

**Keywords:** Motivational interviewing, Outcome measure, Planning, Weight loss maintenance, Behavior change counselling, Lifestyle change

## Abstract

**Objective:**

Understanding mechanisms of motivational interviewing (MI) could improve practice. Planning is important for behavior change, and a component of MI. We aimed to develop a scale to measure planning talk within MI and explore the potential association between planning and weight loss maintenance (WLM).

**Methods:**

A literature review and thematic analysis was used to develop the coding system. Reliability was tested using MI examples and a transcript. It was applied to 50 sessions and associations between planning and WLM were analyzed using logistic and linear regression.

**Results:**

The coding system included: past, continuing, future and hypothetical plans and goal setting, varying on specificity and commitment. The percentage agreement was 86% and 75% for examples and transcript reliability respectively. Frequent planners, potentially decrease their weight 2.8 kg(−9.7, 0.6) and BMI 1.2 kg/m^2^(−3.1, 0.4) more compared to less frequent planners. Frequent goal setters increase their BMI(3.6 kg/m^2^, 1.5, 5.7) and weight (9.5 kg, 3.4, 15.6) compared to non-goal setters.

**Conclusion:**

It is feasible to measure planning, with acceptable agreement. Limited conclusions for the potential associations were demonstrated.

**Practice implications:**

This is the first scale to measure planning, an important aspect of MI which has received less attention.

## Highlights

• Scale developed to measure planning talk within motivational interviewing session.
• It is feasible to measure planning, with acceptable agreement.
• The measure can be applied to data to test associations.

## 1 Introduction

Research indicates that planning has an important role in behavior change [1]. People are more likely to change their behavior if they have made a plan or implementation intention [2]. MI is designed to promote behavior change. It aims to strengthen personal motivation for, and commitment to a specific goal by eliciting and exploring the person’s own reasons for change [3]. There are four key processes within MI; engaging, evoking, focusing and planning (new stage). To date, there is a lack of evidence for specific “active ingredients” that may account for its’ efficacy in relation to these health behaviors [4], [5], [6]. While there has been research looking at how people plan, only two studies have looked at planning within the context of MI [7,8]. There was a medium sized effect of MI on action planning (when, where and how plan [7]) (d = 0.42) [8]. MI interventions can generate statistically significantly more complete action plans than self-administered planning (p < 0.01) [7]. It is important to understand how MI works as this could lead to improvements in practice and efficacy, focus research efforts and facilitate a better understanding of what helps people to change behaviour [9].

There are a number of measures that code client behavior within a MI session: Motivational Interviewing Treatment Integrity (MITI) [10], the Motivational Interview Skills Code (MISC) [11], the Sequence Code for Observing Process Exchanges (SCOPE) [12] and the Client Language Easy Rating (CLEAR) [13]. However these were all developed before the planning stage was added to the MI process, therefore, these measures do not code planning.

Motivational Interviewing (MI) also has potential to improve weight related behaviors [14,15]. One study testing MI intervention found that participants maintained their weight loss (6.1 lb. mean weight difference between groups (P = 0.005) at five years [16]). In order to examine whether planning is an “active ingredient” of MI, weight loss maintenance (WLM) session data was used to test and develop, a coding system for planning. The coding system could also be used to examine the possible association between planning and behavior change. In this study associations between planning and weight loss maintenance (WLM) were explored.

The aim of this study was to develop the planning talk coding system- a scale to measure planning talk within an MI session. We also wanted to test the reliability of this coding system and its ability to code MI session data on the topic of WLM. Finally the coding system was used to explore the association between planning and WLM.

## 2 Method

### 2.1 Data source

The data set used in the development of the coding system and testing of planning was from a weight loss maintenance randomized controlled trial testing an MI based intervention to help people maintain weight loss (WILMA study) [14]. This data consisted of 50 audio recorded MI sessions, where participants consented for their data to be used to examine planning talk. These recordings were over the 6 month intervention face to face period. The number of recordings per participant varied from one to six, with 201 recording in total. We randomly selected one recording per participant for analysis via a formula using excel (except for the 11 cases where there was only one recording).

The WLM data were collected at baseline, 6 and 12 months. The outcome measures used in this analysis were BMI, weight, maintaining a weight loss at 12 months and motivation at baseline. These were measured and calculated as stated below:

1. BMI was measured using calibrated digital scales and a stadiometer using the calculation mass in kg/height (m)^2^.
2. Weight was measured using calibrated digital scales and recorded in kilograms.
3. Maintaining weight loss was coded either yes or no and calculated as follow-up weight minus baseline weight. If the participant’s calculated weight was zero or less they were coded as maintaining a weight loss. If the participant’s calculated difference in weight was greater than zero they were coded as not maintaining a weight loss.
4. Motivation was measured using a Likert scale which captured baseline motivation via the questions; “How motivated do you feel to maintain your weight” with 1 being very motivated and 5 being “not at all motivated”.

### 2.2 Sample size justification

The sample size was determined by the number of participants that had at least one face to face MI session recorded and had given consent for the recording to be used. The sample size for this study was 50 participants.

This sample size provides the precision to estimate an average weight to within 1.109 kg using a 95% confidence interval and assuming a SD of 4 [15].

However, the study was an exploratory study therefore the focus was not on finding statistical significance. The associations were interpreted using 95% confidence intervals (CI) around the effect size.

### 2.3 Development of the planning talk coding system

The development of the planning talk coding system involved two processes, a literature review and thematic analysis of the recorded MI sessions, these informed each other. A systematic literature search was conducted to identify definitions of plans and goals. The definitions formed the basis for development of the coding system. In conjunction with the findings from the literature review, the thematic analysis of recorded MI sessions helped develop definitions of plans and goals and identify different types of plans. Thematic analysis was conducted through group discussion and independent coding. The group members individually coded the MI sessions and then met to discuss the coding and provide feedback at all stages. The group included two psychologists with expertise in behaviour change (SS and RM), a MI expert (SR), a psychologist with counselling training (LC), and a statistician (MK). The definitions identified in the literature were independently applied to four MI sessions by SS, RM and MK, to test whether they fitted the data. LC also independently coded nine MI sessions using the definitions identified in the literature. During this coding all coders also thematically analysed the MI sessions to identify further plans and goals. If the literature definitions fitted they were used to inform the different types of plans and the definition of plans and goals that would be included in the draft coding system. From this coding process the group agreed on the definition of a plan and a goal. They then selected the types of plans that best represented those occurring in the MI sessions. They also identified that the specificity of a plan/goal and the commitment to a plan/goal could also be related to outcome and therefore was incorporated into the coding system. This was based on the finding from the analyses of nine MI sessions as well as relevant literature. From this the draft planning talk coding system was developed.

**Fig. 1:**
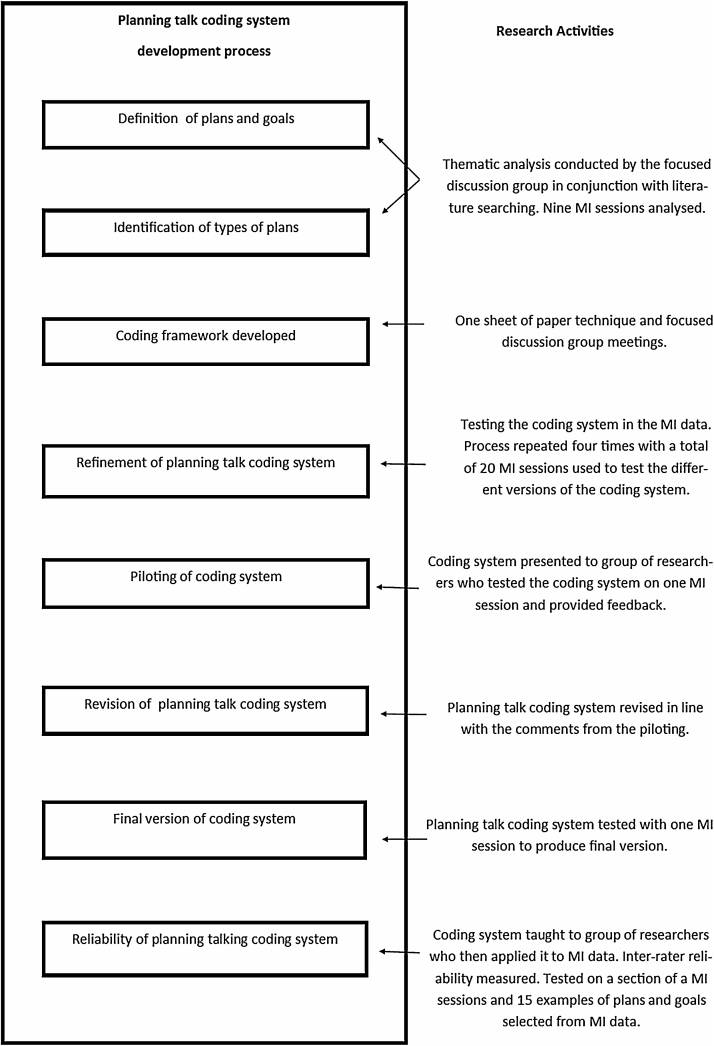
The development of the planning talk coding system.

The coding system was then tested within the MI data and any problems or issues identified were discussed within the group and modifications were made. Each time this occurred, LC tested the revised coding system with five full MI sessions and the group tested it with one of these five sessions. This led to LC coding a total of 20 sessions and from these 20 session SS, RM and MK coded a total of five sessions. A summary of the development of the planning talk coding system can be seen in Fig. 1.

Finally the coding system was tested by a validation group of 11 researchers to check for any further issues. The participants were recruited via an email distributed to different departments within Cardiff University. The group was taught how to use the coding system via a 45 min presentation with examples. The group then applied the coding system to a section of an MI session transcript. Oral feedback was sought from this validation group and used to further refine and clarify the coding system to produce the final version. This process is summarized in Fig. 1.

### 2.4 Reliability of the coding system

The coding system was tested for reliability by a group of 10 Cardiff university staff, who were different from the above researchers (a mix of researchers and administrators). The participants were recruited via an email distributed to different departments within Cardiff University and participants received £40 in vouchers to compensate them for their time.

The participants were trained to use the coding system via a 45 min presentation with examples. They were also given the coding manual and had the opportunity to ask questions. They were then given a transcript of an MI session in which they had to identify the plans and goals. They also coded a list of 15 examples of plans and goals which they had to identify and rate on specificity and commitment. These examples were sections of text that were randomly selected from the MI session data that included either a plan or a goal.

Reliability was measured using a percentage accuracy with a gold standard set by the focused discussion group. The results for each participant, for each exercise was compared for accuracy with the gold standard. A percentage accuracy was the calculated for each participant and an average was taken across all participants A percentage accuracy was used instead of an intraclass correlation to measure reliability for a number of reasons. Firstly, as this was a case of accuracy and not agreement it was not appropriate to use an intraclass correlation. Secondly, an intraclass correlation does not capture the accuracy of participants’ identification of plans and goals at specific lines in the MI session. It only tests the overall numbers of goals and plans identified in the example, which does not capture whether participants identified a plan/goal at a specific line of the MI session. Therefore the ANOVA approach would result in much higher agreement as it would code as agreement at large parts of conversations where no planning talk actually occurred. Instead here, we have summarised accuracy across all raters to identify the accuracy with the gold standard coding. We feel this is a cruder, but ultimately more interpretable summary measure. For these reasons a percentage accuracy was considered a better and simpler representation of the reliability of the coding system.

### 2.5 Planning talk coding system and WLM data

The coding system was applied to the 50 MI sessions by LC, some of which were used in the development process. The frequencies of the different codes were calculated per participant. The variables were trichotomized for analysis into low, medium and high categories (see Table 1). The figures below show how many occurrences of a plan or goal were categorized into the low, medium and high group.

The association between total number of plans made and BMI, weight, and maintaining a weight loss (measured at 12 months) was investigated controlling for individual patient characteristics (i.e. age (18–29/30–59/>59), gender, ethnicity (White/Non-white), source of recruitment (GP practices/Exercise on prescription/Slimming World/Other), percentage weight loss (5–10%/>10%), baseline BMI (30–40/>40), trial arm and motivation).

Graphical illustration (boxplots, histograms and barcharts) was used to explore associations between the different types of plans and weight outcomes. Maintaining weight loss (yes or no) was analyzed using logistic regression, while BMI and weight were analyzed using linear regression.

Inter-coder reliability was used to assess the reliability of the coding of the data. Three independent coders were assigned a random sample of 2 sessions each with an additional one session for two of the coders. Fifteen percent (8) of all sessions were double coded and a percentage agreement statistic was calculated.

## 3 Results

### 3.1 The planning talk coding system

The literature review revealed that there is no uniform definition of a plan or a goal. Many of the explanations of subgoals (a goal that helps you achieve an overall goal e.g. weight loss) were very similar to a plan [17]. Based on the literature review and examples from the data the definition of a plan and goal was agreed.

**Table:** 

Plan: a plan is an action for the future that will help the person achieve a goal and there must be evidence that it was volitional.
Goal setting: a goal is future orientated and is a desired state that the person wants to achieve [18]

**Fig. 2:**
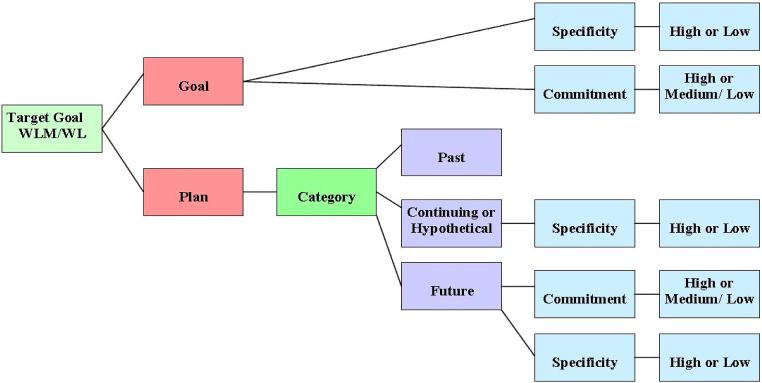
Coding framework for planning talk coding system.

Four additional codes differentiating types of plans also emerged which became part of the planning talk coding system. These were past, continuing, future and hypothetical plans. The definitions of these codes are given in the manual in the appendix. The coding system allows one to first identify if something is a plan or a goal, then if identified as a plan one must choose if it is a past, continuing, future or hypothetical plan (see Fig. 2).

Commitment to a plan/goal was included in the coding system (see Fig. 2) because research has identified that strength of commitment is related to outcome and that it can be measured based on the client’s language [19]. The group agreed that the specificity of the plan/goal should also be included because it has also been shown to be associated with outcome. A meta-analysis [20] found a medium to large association between implementation intentions (when, where and how plans) i.e. more specific plans and goal achievement (effect size d = 0.65).

### 3.2 Reliability of planning talk coding system

Ten participants took part in the reliability testing. They had a variety of research backgrounds both in qualitative and quantitative methods and undergraduate degree backgrounds including English and Psychology. The percentage agreement with the gold standard for plans and goals was 86% for the plans and goals examples and 75% for the transcript reliability respectively.

### 3.3 Planning talk coding system and WLM data

#### 3.3.1 Descriptive statistics

The characteristics of the weight loss maintenance study participants are provided in Table 2.

Initial results indicated that the planning talk coding system did not have any redundant codes. Descriptive statistics of the summary scores showed that all the codes were used during the coding process. There was also a wide range in the numbers of plans made per session from 1 to 39 which demonstrated that the coding system is sensitive to the range of plans made. The median total number of plans made within an MI session was 17 (see Table 3). The type of plan made most frequently was continuing plans with a median of 7 per session. Overall there were more plans than goals made within the MI sessions.

The coding of the MI session was also verified through 3 double coders. The inter-rater reliability between the 3 coders (SS,RM,MK) and the main researcher (LC)was found to be 61.2% percentage agreement. This is moderately good level of agreement.

**Fig. 3:**
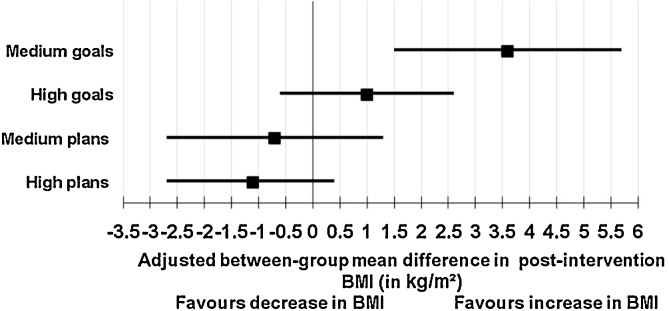
Multiple regression: 95% Confidence interval for categorised goals, plans and BMI.

**Fig. 4:**
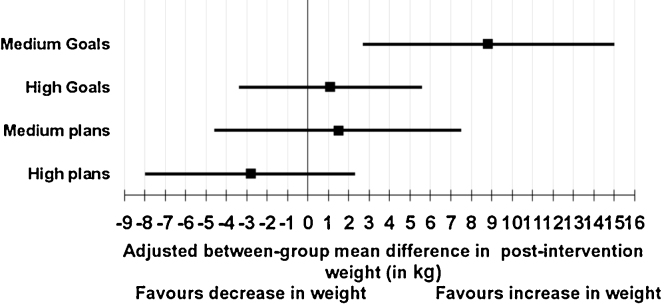
Regression results: regression coefficients and 95% Confidence intervals for categorised goals, plans and weight.

The study is underpowered to detect associations. The results show for high planners a 95% CI −3.1, 0.4 for BMI and 95% CI −9.7, 0.6 for weight. Medium planners are more similar to low planners (adjusted estimate: 1.5 kg, 0.5 kg m^2^ 95% CI). These are not statistically significant results (see Fig. 3, Fig. 4).

Medium goal setters gained 3.6 BMI points (1.5, 5.7) p = 0.001 and 9.5 kg (3.4, 15.6) p = 0.006 compared with non-goal setters and this was statistically significant (see Fig. 3, Fig. 4).

**Fig. 5:**
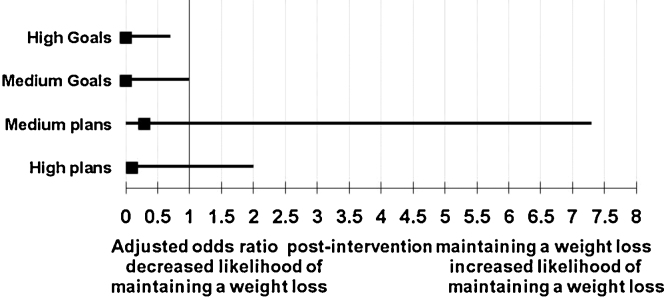
Multiple regression: Odds ratio for categorised goals, plans and maintaining a weight loss.

When comparing high planners to low planners the result was not statistically significant. Investigating goals there is a statistically significant association between high and medium goal setters and maintaining a weight loss. They are less likely to maintain a weight loss compared to those who set no goals (see Fig. 5).

## 4 Discussion

Although planning has been identified as an important aspect of MI, to date although there are validated measures of other aspects of MI sessions, there is no measure of planning that has been developed to code plans within MI sessions. This new coding system shows potential as a measure for coding planning talk.

The results from the reliability of the coding system demonstrated a good level of reliability. The examples led to an 86% agreement and the transcript led to a 75% agreement with the gold standard. This is similar to the SCOPE and the MITI inter-rater reliability results that were in the good to excellent range [21,22]. The lower agreement levels of the transcript scores could be explained by the fact that the transcript needed to be parsed. Within other measures this has been identified as a subjective process and therefore possibly unreliable [23]. Parsing is an area to be improved for future training and reliability. Nevertheless this study has shown that non-experts can be trained in a brief training session to apply the coding system and that the results they produce are highly reliable.

The study was underpowered to detect any associations. The testing of an association between planning and WLM outcomes demonstrates how the coding system could possibly be used and the type of result one might obtain. To test the association between planning and BMI and weight we need to test within a larger data set.

Medium goal setters statistically increase both their BMI and weight. The results suggest that goals were not associated with a decrease in weight. This is an unexpected finding but may not hold up in other/larger samples and it would be helpful for future studies to look at interaction effects. It may also be important to make plans alongside goals and not set goals without articulating a clear plan to achieve them. It may also be that goals are not specific enough (i.e. not as specific as plans) and therefore not sufficient. This finding appears contradictory to evidence suggesting that goal setting can help people change their behaviour [17,18]. However the evidence around planning does suggest that there is a long road between goal intentions and achieving them, as people need to deal with repeated interruptions and possible setbacks [24]. It may be that goals are necessary but not sufficient to lead to behaviour change, i.e. plans are what you need to achieve your goal. In the current study however, we were unable to examine this hypothesis as the data does not allow for plans to be linked to specific goals (i.e. it was not possible to fit an interaction term). This is a potential area for future research.

This study has a number of strengths mainly the iterative nature of the development of the coding system and independent corroboration of the final coding system. The coding system was developed using inductive thematic analysis (applying codes from the literature) [25] and the deductive approach (using the MI sessions to develop the codes) [25,26]. Combining both these approaches led to insights from the MI data which were combined with theory and published evidence.

The coding system was also developed via a rigorous process of testing the coding system within 20 MI sessions. This process was similar to procedures used in the development of the Behavior Change Counselling Index (BECCI) [27] (25 sessions) and the Evaluation of AGenda mapping skiL Instrument (EAGL-I) [28] (35 sessions). The MI intervention was also delivered with high levels of fidelity [14] meaning the coding system was based on and tested within sessions where MI was actually delivered.

Good reliability of the coding system was demonstrated after training a group of 10 researchers and applying the coding system to a section of the MI session data. These results are similar to other studies that have used the MISC or the MITI which found an inter-rater reliability score of between 0.59 and 0.95 [29], [30], [31], [32].

The researcher (LC) was blinded to the weight maintenance results. Therefore the coding of the MI data using the planning talk coding system was not influenced by any knowledge of the participant’s weight results. The coding of the 50 MI sessions using the coding system were also double coded by 3 researchers with an average percentage agreement of 61.2% which is a good/acceptable level of agreement.

There are a number of limitations to this study. Firstly the coding system was developed and tested for reliability on WLM data so it may not be generalizable to other data sets. However, the codes within the coding system have been developed to be general and not specific to weight loss or weight loss maintenance data. Further research needs to be conducted to test the reliability of the coding system within other MI data sets with different outcomes.

As this study was underpowered it limited the statistical interpretations that could be drawn from the data. The results need to be tested within a larger data set to assess whether the current findings could be replicated. In addition, the results should be interpreted with caution as further development of the planning talk coding system is needed. This includes looking at the internal consistency, stability and responsiveness of the tool: these issues need to be investigated to ensure the reliability of the measure.

### 4.1 Conclusions

In conclusion, while caution is advised with the interpretation of the association between planning and outcomes due to sample size as well as the stage of development of the coding system, these findings are encouraging as it is possible to measure planning within a MI session and that it is potentially associated with behaviour change and a mechanism of MI. There is also some evidence that the total number of plans is an important factor for successful weight maintenance.

### 4.2 Practice implications

To date, there is a lack of evidence for specific “active ingredients” that may account for the efficacy of MI in addictions and health behaviours [22,24]. Understanding the mechanisms affecting outcomes could lead to improved practice and planning is a candidate mechanism. The planning talk coding system makes it possible to code this. This is useful to researchers as they can measure planning and examine associations with relevant outcomes thus increasing our understanding of potential mechanisms in MI. A group of mixed group of researchers and administrators were trained to use the coding system within 2 h, and our results demonstrate that the coding system can be used reliably by this group. As the coding system can be used following minimal training, it can easily be used in a research or practice environment without incurring significant resource.

Understanding planning within MI could improve practice, as therapists could use the findings from this research to change the way they work in two ways. Firstly, the results from this study suggest it is important for clients to make plans in order to improve the chances of behaviour change happening. Therefore it is important for therapists to recognise that planning is a potential key element of MI and to ensure they facilitate individuals to make plans to achieve behaviour change. Secondly this measure could be used in routine practice during supervision or in MI training to assess how well practitioners are facilitating planning phase.

## Figures and Tables

**Table 1:** Number of plans and goals for trichotomized variables.

Plan/Goal variable	Low	Medium	High
Medium plans	0–13	13–20	21+
Medium goals	0	1–3	4+
Medium future plans	0–2	3–5	6+
Medium continuing plans	0–5	6–8	9+
Medium Hypothetical plans	0	1	2+
Medium Past plans	0–2	3–4	5+
Medium high commitment plans	0	1–3	4+
Medium low commitment plans	0	1–3	4+
Medium high specificity plans	0–6	7–12	13+
Medium low specificity plans	0–2	3–5	6+
Medium high commitment goals	0	1	2+
Medium low commitment goals	0	1	2+
Medium high specificity goals	0	1	2+
Medium low specificity goals	0	1	2+

**Table 2:** Participant characteristics.

Baseline measures	Mean	SD
Age (years)	49.9	12.3

Baseline measures		
Weight (kg)	90.4	19.5
Height (cm)	164.9	7.2
Waist (cm)	102.2	16.2
Hip (cm)	117.7	12.4
BMI (kg/m2)	33.2	6.3

Gender		
Male	14% (n = 7)	
Female	86% (n = 43)	

Ethnicity		
White British	90%	
White Irish	2%	
Other white background	4%	
Other	4%	

**Table 3:** Descriptive statistics of summary scores for each type of plan within the coding system.

Planning talk coding system data		
Type of plan or goal	Median	IQR
**Total No. Plans**	**17**	13–22.5

**Future Plans**	**5**	**3–7**
Future plans High Commitment	3	1–4.25
Future Plans Low Commitment	2	1–4
Future plans High Specificity	3	2–4
Future plans Low Specificity	1	0–3

**Continuing Plans**	**7**	**4–11**
Continuing Plans High Specificity	6	3–8.25
Continuing Plans Low Specificity	2	1–3

**Goal Setting**	**2**	**1–4**
Goal Setting High Commitment	1	0.75–3
Goal Setting Low Commitment	1	0–1
Goal Setting High Specificity	1	0–1
Goal Setting Low Specificity	1	0.75–3

**Hypothetical Plans**	**0**	**0–1**
Hypothetical Plans High Specificity	0	0–1
Hypothetical Plans Low Specificity	0	0–0

**Past Plans**	**3**	**2–6**
Total high commitment plans	3	0–7
Total low commitment plans	2	0–7
Total high specificity plans	10	2–23
Total low specificity plans	4	0–15
