# Supplementary material for: The development of a coding system to code planning talk within motivational interviewing
Source: Patient Educ Couns. 2017 Feb;100(2):313–9. doi: 10.1016/j.pec.2016.09.003 (PMC5332025; doi:10.1016/j.pec.2016.09.003)
Supplement: Supplementary file 1 [file mmc1.docx]

**Appendix C4-3 Planning talk coding system manual**

**Coding System**

**Conceptual model**

**Definition of a plan**

A plan is an action for the future, it is volitional, involves thought before action and contains behaviours. Planning refers to the development of specific alternative behavioural paths by which a goal can be attained (Austin & Vancouver , 1996). They decide on a behavioural action, which is the ‘‘how’’ they will achieve the goal (Sniehotta, Scholz and Schwarzer, 2005). The “how” is the essential part of planning, the “when” and the “where” of a plan is optional for the definition of a plan, but does help to make the plan more specific. It can also involve details of actions or strategies to help the person negotiate potential obstacles (Sniehotta, Schwarzer, Scholz and Schu, 2005). If there is evidence of volition and thought before action from what the client has said then it is planning. This evidence may come in the form of the client informing the clinician of an activity they are doing or commenting on how an action is going.

Examples of plans are:

C: “You, you don’t have to go up to to fifteen sins, every sin is twenty calories and so I suppose that’s three hundred so you’re allowed calories of nice things, you know, chocolate, chips, whatever um but I I, you you there’s a pl... ((woman’s name)), lovely, marvellous um person who runs the club, says you have to learn to manage them. If you cut them out completely, you cannot go for the rest of your life never eating these things. You have, you have to tame them, yes? You control them.”

P: “So you tame the the (bits), yeah”

C: “Tame them and you manage them. Don’t let them manage you.”

P: Hmm.

C: “So what I, so what I must do this week, is try and tame these things.”

P: Ok. So that sounds like a really clear target.

“I’m going to eat breakfast every day.”

“Uuum yeah I think we talked about it the night before that we were going to go shopping a

bit later than originally planned.”

**Definition of a Goal**

A desired state that a person wants to achieve, where states are outcomes, events, or processes. (Austin & Vancouver , 1996). This desired stated must be in the future. These desired states could range from internal processes (e.g. to be less stressed), to desired outcomes (e.g., career success). The desired stats are indicated by the client using words such as “want”, “aim”, “desire”, “aspire”, “achieve” and “longing” etc. The goals will be states that the client wants to achieve that will help them reach the overall target goal. The goal is therefore contingent upon the performance of actions to achieve it.

Example of a goal are:

“I want to lose a pound a week”

“I want to go to the gym twice a week”

**The difference between a Goal and a Plan.**

The difference between plans and goals is determined by the “How” element of “How”, “When” and “Where”. The “how” is the essential part of planning that defines it, the “when” and the “where” of a plan is optional for the definition of a plan. The goal, however does not include the “How” element. Therefore if there is just a desired state mentioned then it is a goal. A goal and a plan can occur adjacent to each other within the same sentence.

**Identifying the Target Goal**

MI is used to identify and encourage change talk and to specify how these changes will take place therefore the therapist must have the target goal in mind, helping them to know which instances of client language to focus on. Before coding the session the coder should be made aware of the target goal. The target goal is overall what the client wants to achieve and is long term in nature. This target goal must always be kept in mind when coding the session and it must be specified in enough detail to allow the coder to discriminate it from other topics the client may talk about. There may be many behaviours that are linked to the target goal that can be included in the coding.

Examples of target goal are:

Weight Loss (target goal)

Weight Loss Maintenance

Stopping smoking

Increasing Fruit and vegetable intake

Adhering with medication regimen

Not stress eating

Coders should not infer a link between actions and the target goal unless it’s clear from the context that the clients plan will help them move towards their target goal. For example “I plan on being less stressed work” would not be a plan towards a target goal of weight loss. However, if this was linked to stress eating then it would be. It would also be linked to the target goal if the therapist mentions the target goal and in response the client mentions a plan.

**Assigning codes**

P: Past Plan

C: Continuing Plan

F: Future Plan

H: Hypothetical Plan

GS: Goal Setting

Everything is coded on a degree on specificity: Low or High with the exception of past plans. With the exception of Hypothetical plan, Past plan and Continuing plan, everything is coded on a degree of commitment: Medium/Low or High.

**Repetition of Plan or Goal**

If the same plan/ goal is mentioned more than once throughout the session it is coded once only if it could be assigned two codes from the same category, such as both assigned at future plan code. This follows the MITI 3.1 as they state “Once a behaviour code is assigned once within the volley, it is not assigned again. A volley may contain only one of each behaviour code”. The plan/goal that is coded is the plan/ goal with the highest commitment and specificity score assigned to the plan/ goal throughout the session. All other instances of the same plan/ goal that are mentioned and can be coded with the same category such as future plan are not coded. **However**, if the plan/ goal if retracted during the session then the plan/ goal is no longer coded.

For example

C: Well, my son’s birthday is Monday…

P: Yeah.

C: and we’ll be having a tea party although that’s good cos it’s on Monday, I’ll be going to Slimming World at seven o’clock regardless to get weighed

P: Right.

C: even if I don’t stay so that’ll make me…

P: Ok.

C: **perhaps I’ll have the piece, slice of birthday cake when I get home, then…** ((laughter))

P: give you an incentive…

C: **and won’t have it until the middle of the party.** (future plan with high specificity and low commitment **BUT NOT CODED** as future plan repeated later in conversation) ((Phone rings)) Oh I’ll ignore that …

P: Yeah?

C: Yeah.

P: Ok. Yeah, so it’s a bit of an incentive for you not to uh not to do that at the party then.

C: Yeah.

P: Yeah. So you feel like you have some form of a plan to deal with the food at your son’s birthday. Sounds like you are going to try and have only a small amount of cake.

C: **Yeah, I will definitely only have a small slice of cake after I come back from being weighed at slimming world. Slimming world will give me the motivation I need to stay on track with my diet. (** CODED as Future plan with high specificity and high commitment).

The second time the future plan about cake is coded as this has been assigned the highest commitment and specificity code. The first time the future plan is mentioned it is not coded.

However if the same plan is repeated but can be assigned two different categories of plan then both times the plan is stated will be coded.

For example

If I go for a walk everyday then I would have to leave work early (hypothetical plan- **coded**), so I’m definitely going to start doing that (future plan- **coded**)

**Client responses to clinician questions.** Clients may respond to clinician questions/ statements with language that fits within any of the planning talk categories, and it should be coded as such. The fact that the clinician “set it up” with a particular sort of question or comment does not mean that the client’s response is not planning talk. Even a one-word answer to a question may qualify for a planning talk code if the coder deems it to be a genuine response rather than simply a socially facilitating response. For Example:

Counsellor: So your planning on going to the gym more often specifically every Monday night after work and you are gonna bring your gym close with you to work.

Client: Yes that’s right.

**Definitions of Codes**

**Past Plan**; These plans will consist of an expression of an action that has happened or a circumstance that previously existed. It must be a volitional plan that was made about a future action that has now been completed. There also must be evidence from what the client has said that there was volition or evidence of justification for the plan. Volition cannot be implied. To be coded the behaviour must clearly be one that is intended by the client to lead to the target goal. For example:

“I made pasta last week and instead of making a cheese sauce I made a tomato sauce so I didn’t use my calorie allowance”

“I went to the gym last week with a friend from work”

**Continuing plan**: This is a strategy to continue the same action or event at a repeated frequency either continuously or at intervals. The plan has been made before the therapy session and will continue to be enacted in the future. To be coded the behaviour must clearly be one that is intended by the client to lead to the target goal. For Example

“When I’m at home I only snack on vegetables and that’s something I’m gonna keep up”

“I’ve been making a packed lunch for work every day and I’m gonna make sure I keep doing that”

“I decided to take up knitting to distract myself from my craving which is really helping so I want to keep that up”

“I will walk everyday to work like I have been going as that really helps”

**Future Plan**: Is a plan that will or is likely to happen in the period of time after the therapy session. It must be a volitional plan that was made about a future action that has not been completed yet. To be coded the behaviour must clearly be one that is intended by the client to lead to the target goal. For example

“I’m going to start going to the gym”

“I’m going to eat fruit every morning for breakfast”

**Hypothetical**: Is an idea about what the client could do to move towards the target goal but that the client is not committing to. A client could use the hypothetical to problem solve possible barriers they may come against. This must be an idea that could help them move towards the target goal or a way of moving past barriers that could stand in their way of completing the target goal. For Example:

“If I cut out pasta I could lose more weight”

“If I forget my gym kit I could go for a walk on my lunch break”.

“If I make my lunches for work I think it could help me loss weight”

**Goal Setting**: An internal representation of desired states, where states are broadly construed as outcomes, events, or processes. (Austin & Vancouver , 1996). This desired stated must be in the future. Internally represented desired states range from internal processes (e.g. to be less stressed), to complex cognitive depictions of desired outcomes (e.g., career success). The desired stats are indicated by the client using words such as “want”, “aim”, “desire”, “aspire”, “achieve” and “longing” etc. The goals will be states that the client wants to achieve that will help them reach the overall target goal. The goal is therefore contingent upon the performance of actions to achieve it. **The goal does not include a statement of what the actions are to achieve it (the “How”) as that defines a plan.** When coding goal setting be aware that when the word “if” occurs before a goal e.g. “If only I could be 10 stone” this is not coded as a goal. The client is not stating a goal they want to achieve.

“I want to lose a pound a week”

“I want to go to the gym twice a week”

**Rating the Degree of Commitment**

Every time Future or Goal Setting are coded a degree of commitment must be assigned: Medium/Low or High. Hypothetical does not get assigned a degree of commitment as the nature of this implies the clients are not committed. Past also does not get assigned a degree of commitment as due to the nature of the language used when people talk about past plans one would not be rating the degree of commitment to the plan but the success or failure of the plan. A person can be very committed to a plan but unforeseen events may stop the person completing the plan counting it as a failure. Continuing is also not rated on commitment as the plan is continuing to happen. Degree of commitment implies an intention or an obligation to complete the plan/ goal made by the client. The client is pledging to complete a certain behavioural act. This includes starting a certain behaviour as well as stopping something, such a giving up chocolate. This can be expressed directly via a committing verb, or indirectly. For example:

“I *swear* I will stop this” “Swear” is strong committing verb, coded High

“Nothing is going to stop me this time” This statement has no committing verb, but it indirectly implies commitment and is also coded High

There are separate commitment ratings scales for plans and goals as plans are about going to an action where are goals are about wanting to achieve something. These lead to different commitment verbs being used.

**Commitment rating for plans**

**High:** Statements should indicate that the client is going to complete the plan made. It should indicate that they have strong intentions to change their behaviour.

**High Commitment Words**

| I guarantee  I will  I promise  I vow  I shall  I give my word  I assure  I dedicate myself | I know  I am devoted to  I pledge to  I agree to  I am prepared to  I intend to  I am ready to  I’ve got to  I know I can  I’ll do  I definitely will  I’m going to  I just got to  I’m not going to  I’m definitely not going to  There is no way |
| --- | --- |

“Well, I I know now, I’ll be more structured”- Future

“I took my portion sizes um and I need to get myself organised a bit more with my food

know what I’m having for the following day and get it prepared.”- Future

“I’d love to get it off to go this wedding”- Goal setting.

**Medium/ low:** Statements should indicate that the person may complete the plan however they have not fully pledged to change their behaviour. They may also be wanting to see how things go and haven’t fully made up their mind.

**Medium/ Low Commitment Words**

| I look forward to  I consent to  I would like to  I plan to  I resolve to  I expect to  I concede to  I declare my intention to  I favor  I endorse  I believe  I accept  I volunteer  I aim  I aspire | I propose  I am predisposed  I anticipate  I predict  I presume  I mean to  I foresee  I envisage  I assume  I bet  I hope to  I will risk  I will try  I think I will | I suppose I will  I imagine I will  I suspect I will  I contemplate  I guess I will  I wager  I will see (about)  I could do  Perhaps I could  Hopefully I can  Maybe I  I think  I might not  Possibly I will not |
| --- | --- | --- |

“I think for the moment, I’d want to go back to weighing”- Future

“So maybe that’s what I need to step up a bit” –Future

“Yes, and plan ah… and plan, yeah, factor in the whole day, I suppose” -Future

**Commitment scale for goals**

**High**: Statements should indicate that the client is very committed to the goal, wants to achieve it or shows how important the goal is to the client. This can be expressed through the client language or through the client rating on a scale of 1 to 10 how important the goal is. If the client rates the goal as a 6 or higher then is it is rated as high commitment.

**High Commitment Words**

| I really want  I truly want  I desperately want to  I definitely want to achieve  I want to | I would love to  I absolutely want to  I undeniably want to  It’s really important  I have to  I definitely don’t want to  It’s very important I don’t |
| --- | --- |

“I definitely want to lose 1 stone”

“I truly want to eat healthier”

**Medium/Low**: Statements should indicate that the client is not as committed to the goal and is less important to the client. This can be expressed through the client language or through the client rating on a scale of 1 to 10 how important the goal is. If the client rates the goal as 1-5 then is it is rated as high commitment.

**Medium/Low Commitment Words**

| I would like to  I kind of want to  I might want to  Maybe I would like to | I want to possibly  I suppose |
| --- | --- |

“I’d like to get to about twelve stone six, I would”

**Rating the Degree of Specificity**

Every time, Continuing, Future, Hypothetical or Goal Setting are coded a degree of specificity must be assigned: Low or High. Past plans are not rated on specificity. There are separate specificity rating scales for plans are goals are they comprise of different components and does not include a “how”. Degree of specificity implies a clearly defined set of events that precisely identify when and how and possibly where an action will take place.

**Specificity rating for plans**

**High**: Statements must include “when” a time scale to achieve it in, the time the plan will occur or it could be a cue, a state of being or a circumstance e.g being stressed or when they are at work. It should also include “how” they are going to achieve what they want. “Where” the plan takes place can be included as well but will not lead to the plan being rated as high specificity without the presence of “when”. The wording of the “when” should be more specific than “sometime”, “at some point” or “in the future” for it to be coded as the “when” of a plan. If however the client says “this year” or “this summer” etc then that is coded as “when”. If a client says that they are never going to do something again/ has stopped doing something or is never going to go back to a certain state that is also coded as “when”.

“I took my portion sizes um and I need to get myself organised a bit more with my food.

Know what I’m having for the following day and get it prepared”- Future

C: Like like in the night then to watch my portion, I’ll weigh my rice ready...

P: Ok.

C: and things. Put it in a saucepan ready so when I come in from work or anything, there’s

no looking for what I’ve got to have. -Future

“If I could eat carrots instead of biscuits at my 3pm tea break at work then I could lose some weight” -Hypothetical

P:Ok how else did you lose this weight?

C:Um, ex, well I uh line dance three times a week.

P:Wow.

C: and that’s usually um an hour and a half. It’s always like a two hour

session but if you have a coffee or a tea or if you’re there a bit late, but I’m always dancing for an hour and a half…

P:Hmm mm

C:and that’s three times a week. - Continuing

**Low**: Statements must include “how” the client is going to achieve what they want. “Where” the plan takes place can be included as well but will not lead to the plan being rated as high specificity. Statements that include the “When”, a time scale to achieve it in/ the time the plan will occur, should be rated as high.

“I’m going to eat more fruit” Future plan

“I’m going to continue to be more active” Continuing plan

“Well, I I know now, I’ll be more structured”- Future

**Specificity rating for goals**

**High**: Statements for rating goals as high must include “when” the goal should be achieved by. The wording of the “when” should be more specific than “sometime”, “at some point” or “in the future” for it to be coded as the “when” of a plan. If however the client says “this year” or “this summer” etc then that is coded as “when”. “Where” the goal takes place can be included as well but will not lead to the goal being rated as high specificity without the presence of “when”.

“I want to lose 1 stone by my holiday”

“I want to start going to the gym on Mondays”

“I want to eat fruit daily”

**Low**: Statements for ratings goals as low will only include the goal that the person wants to achieve. “Where” the goal takes place can be included as well but will not lead to the goal being rated as high specificity.

“I want to lose 3 pounds”

“I want to go to the gym”
